# Efficacy of two-week therapy with doxycycline-based quadruple regimen versus levofloxacin concomitant regimen for helicobacter pylori infection: a prospective single-center randomized controlled trial

**DOI:** 10.1186/s12879-021-06356-5

**Published:** 2021-07-04

**Authors:** Marouf Alhalabi, Mohammed Waleed Alassi, Kamal Alaa Eddin, Khaled Cheha

**Affiliations:** Gastroenterologist at Damascus Hospital, Almujtahed street, Damascus, Syria

**Keywords:** Helicobacter pylori, Doxycycline, Tinidazole, Bismuth, Quadruple regimen, Levofloxacin, Syria

## Abstract

**Background:**

Antibiotic-resistance reduces the efficacy of conventional triple therapy for Helicobacter Pylori infections worldwide, which necessitates using various treatment protocols. We used two protocols, doxycycline-based quadruple regimen and concomitant levofloxacin regimen. The aim was to assess the effectiveness of doxycycline-based quadruple regimen for treating Helicobacter Pylori infections compared with levofloxacin concomitant regimen as empirical first-line therapy based on intention-to-treat (ITT) and per-protocol analyses (PPA) in Syrian population.

**Settings and design:**

An open-label, randomised, parallel, superiority clinical trial.

**Methods:**

We randomly assigned 78 naïve patients who tested positive for Helicobacter Pylori gastric infection, with a 1:1 ratio to (D-group) which received (bismuth subsalicylate 524 mg four times daily, doxycycline 100 mg, tinidazole 500 mg, and esomeprazole 20 mg, each twice per day for 2 weeks), or (L-group) which received (levofloxacin 500 mg daily, tinidazole 500 mg, amoxicillin 1000 mg, and esomeprazole 20 mg each twice per day for two weeks). We confirmed Helicobacter Pylori eradication by stool antigen test 8 weeks after completing the treatment.

**Results:**

Thirty-nine patients were allocated in each group. In the D-group, 38 patients completed the follow-up, 30 patients were cured. While in the L-group, 39 completed the follow-up, 32patients were cured. According to ITT, the eradication rates were 76.92%, and 82.05%, for the D-group and L-group respectively. Odds ratio with 95% confidence interval was 1.371 [0.454–4.146]. According to PPA, the eradication rates were 78.9%, and 82.05% for the D-group and L-group respectively. The odds ratio with 95% confidence interval was 1.219 [0.394–3.774]. We didn’t report serious adverse effects.

**Conclusions:**

Levofloxacin concomitant therapy wasn’t superior to doxycycline based quadruple therapy. Further researches are required to identify the optimal first-line treatment for Helicobacter-Pylori Infection in the Syrian population.

**Trial registration:**

We registered this study as a standard randomized clinical trial (Clinicaltrial.gov, identifier-NCT04348786, date:29-January-2020).

## Background

Eastern Mediterranean region countries have a high prevalence rate of Helicobacter Pylori (H.Pylori) infection [1]. Chronic infection of *H. pylori* contributes to multiple diseases such as peptic ulcer disease and subsequent bleeding [2–4], dyspepsia, gastric adenocarcinoma [5], mucosa-associated lymphoid tissue (MALT) lymphoma [6], idiopathic thrombocytopenic purpura [7], and unexplained iron deficiency anaemia [8]. World health organization has listed the H.Pylori infection as a class 1 carcinogen [9]. Eradication of H.Pylori cures previous diseases and can decrease the risk of gastric cancer [10]. Eradication rate of *H. pylori* infection is declining globally due to increased antibiotic resistance particularly clarithromycin and levofloxacin [11]. In the eastern Mediterranean area, the resistance to clarithromycin, metronidazole, levofloxacin, amoxicillin, and tetracycline were 29%, 61%, 23%, 14%, 10% respectively [12]. Several researchers reviewed many therapeutic regimens including sequential, concomitant, and hybrid to find the best treatment protocol [13]. The results of conventional triple therapy in Syria were disappointing [14]. Currently tetracycline is unavailable in Syria, so we used doxycycline in the bismuth quadruple regimen [15, 16]. Although tinidazole isn’t superior to metronidazole in treating Helicobacter Pylori infections [17], we used tinidazole instead of metronidazole in both regimens, as *H. pylori* had high metronidazole resistance rate [18]. Metronidazole is a commonly overused drug in Syria, mainly prescribed for gynaecological and gastrointestinal diseases [19] Besides, tinidazole (b.i.d) is more tolerable by patients. There is a lack of data about the efficacy of doxycycline-based quadruple regimen and levofloxacin-containing quadruple concomitant regimen in Syrian patients, we conducted this trial to evaluate the efficacy and report the eradication rate of these regimens according to Intention-to-treat analysis (ITT) and per-protocol analysis (PPA).

## Methods

This was a prospective single-center open-label parallel randomized superiority controlled clinical trial. It was conducted at gastroenterology department, Damascus hospital, Syria. We recruited appropriate candidates from patients who visited our clinic for evaluation of dyspeptic symptoms by upper gastrointestinal endoscopy between February 2020 and August 2020. Exclusion criteria were (1) younger than 18 years and older than 80 years; (2) prior eradication treatment for H pylori; (3) documented reactions to any of the studied medications;(4) recent use of antibiotics, bismuth, or proton pump inhibitors (PPIs) in the preceding month; (5) pregnant or lactating women; (6) previous gastric surgery; (7) alcohol or opioid abuse; and (8) severe concurrent medical illnesses, such as liver failure, renal failure, or terminal malignancy.

### *H. pylori* detection

All patients have undergone upper gastrointestinal endoscopy. Endoscopists have taken five gastric biopsies; two from the antrum, two from the body, and one from the incisura according to the Sydney system [20]. Pathologists confirmed *H. pylori* infection by microscopic examination after using haematoxylin, eosin, and Giemsa stains [21]. We sent all biopsies to the central pathological laboratory of the same referral hospital.

### Intervention

Eligible patients were randomized in a 1:1 ratio to receive 2 weeks of treatment of either doxycycline-based regimen (D-group) or concomitant levofloxacin regimen (L-group). The D-group obtained bismuth subsalicylate 524 mg q.i.d, doxycycline 100 mg, tinidazole 500 mg, esomeprazole 20 mg each b.i.d for 14 days. While the L-group obtained levofloxacin 500 mg q.d, tinidazole 500 mg, amoxicillin 1000 mg, and esomeprazole 20 mg each b.i.d for 14 days. The indication of treatment relied on the American College of gastroenterology guideline and Maastricht V/Florence consensus report [13, 15] including peptic ulcer, chronic gastritis, primary gastric MALT lymphoma, intestinal metaplasia, dyspepsia, and unexplained iron deficiency anaemia.

We used a Microsoft Excel function called (RANDBETWEEN) to generate a sequence of two randomized numbers, number one referred to the D-group, and number two referred to the L-group. We printed each code on separate paper, inserted it into sealed opaque envelopes in unchanged order, and hold it in a secure locker belonging to an independent medical staff member. After obtaining informed consent, the independent medical staff member took the top envelope to assign the patient to the treatment regimen. We provided all patients with written instructions considering medication dosage. We evaluated compliance by counting the number of unused medications and considered that the patient was complaint if he/she had taken at least 90% of the assigned treatment protocol.

At the end of the treatment course, patients revisited the clinic to investigate side effects and evaluate compliance. We reported side effects such as nausea, vomiting, diarrhoea, melena, dysgeusia, and anorexia. After 8 weeks, all patients visited the central laboratory of our hospital and undergone stool antigen tests by using the enzyme immunoassay method (EIA) [22]. Medical laboratory workers were blinded to the treatment arm. Qualified physician collected the data in a questionnaire including (1) participants’ demographics; (2) smoking history; (3) medication history; (4) adverse events, and (5) results of stool antigen test. Numerical data were shown as mean, and qualitative data were expressed as a ratio.

Authors reported the results according to the CONSORT.

### Outcomes

This study aims to assess the effectiveness of doxycycline-based quadruple regimen for treating Helicobacter Pylori infections compared with levofloxacin concomitant regimen.

### Sample size and statistical analysis

We reviewed English medical literature for Helicobacter Pylori doxycycline-based therapy and found a systematic review authored by Niv [16] .There aren’t new studies yet. We reviewed the studies within it and found that nine clinical trials excluded bismuth, two clinical trials replaced proton pump inhibitors with ranitidine, and two clinical trials used different doxycycline therapeutic protocol (LOND: levofloxacin, omeprazole, nitanoxanide, and doxycycline) [23]. The remaining two studies: Borody TJ et al. reported the eradication rate of doxycycline based therapy as first line treatment and it was 0.65 [24],while Wang et al. reported the eradication rate of doxycycline based therapy as second-line treatment based on ITT, It was 0.6744 [25]. Those results were close and we chose the highest eradication rate. Federico et al. found that the eradication rate based on ITT was 0.922% in concomitant levofloxacin-containing therapy [26]. To find if concomitant levofloxacin-containing regimen was more effective than doxycycline-based regimen we conducted a clinical trial with superior study design. We used a power (1-β) of 80%, two tails test and significance level (α) equal to 5%, with a 1:1 allocation ratio. Each treatment arm required 37 patients [27]. We added two patients to each group to compensate for the predicted dropout [28, 29].

Statistical tests: Chi-square test (χ2 -test) for categorical variables, and t-test for continuous data. We reported the odds ratio with a 95% confidence interval. A *P*-value less than 0.05 was considered statistically significant. We performed statistical analyses using SPSS (IBM Corp. Released in 2017. IBM SPSS Statistics for Windows, Version 25.0. Armonk, NY: IBM Corp).

## Results

**Fig. 1:**
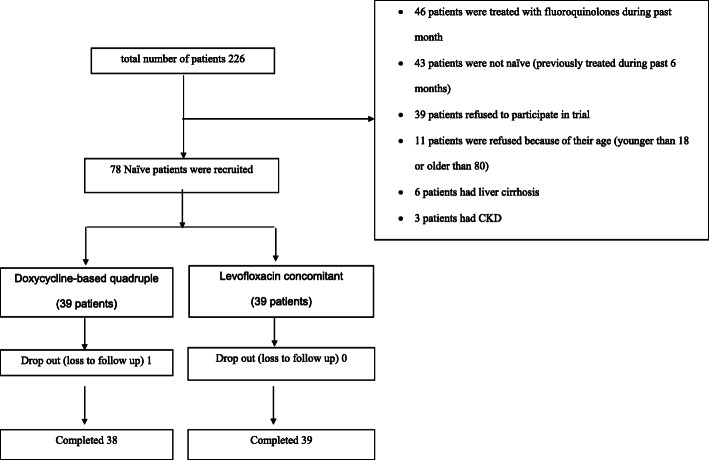
Flow of the study

We collected 226 patients diagnosed with *H. pylori* infection confirmed by biopsy. Seventy-eight treatment-naive patients were enrolled in this study (39 patients for each group), only one patient in D-group didn’t complete the follow-up. [Fig. 1 and Table 1] summarizes the flow chart and baseline characteristics of the patients. The gender, mean age and pharmacological side effects were similar among treatment groups, except for melena, which occurred more frequently in the D-group.

**Table 1:** Baseline characteristics of patients

	doxycycline-based quadruple regimen	levofloxacin concomitant regimen	*P*-value
Gender			
Male	21 (55.3%)	20 (51.3%)	0.821
Female	17 (44.7%)	19 (48.7%)	
Age (mean years ±SD)	41.82 ± 11.914	45.62 ± 17.994	0.223
Smoking	16 (42.1%)	16 (41%)	1.00
Alcoholic	2 (5.3%)	0 (2.5%)	0.240
**Adverse events**			
Anorexia	5 (13.2%)	11 (28.2%)	0.160
Nausea	10 (26.3%)	5 (12.8%)	0.160
Vomiting	7 (18.4%)	4 (10.3%)	0.347
Dysgeusia	7 (18.4%)	11 (28.2%)	0.421
Melena	11 (28.9%)	0 (0%)	≤ 0.0001
Discoloured Tongue	1 (2.6%)	0 (0%)	0.494
Diarrhea	6 (15.8%)	3 (7.7%)	0.310

*H. pylori* infection was eradicated in 30 patients from the D-group, and 32 patients from the L-group. Table 2 summarised the eradication rates according to ITT and PPA analysis. We didn’t report serious adverse effects.

**Table 2:** Helicobacter pylori eradication rate

	levofloxacin concomitant regimen	doxycycline-based quadruple regimen	Odds Ratio (95% CI)	*P*-Value
ITT	82.1%	76.9%	1.371 (0.454,4.146)	0.78
PPA	82.1%	78.9%	1.219 (0.394,3.774)	0.78

*CI* confidence interval, *ITT* intention to treat analysis, *PPA* per-protocol analysis

## Discussion

Researchers globally observe a decline in the rate of H pylori eradication following standard triple therapies, thus requiring a search for new therapeutic approaches [45–48]. This randomized clinical trial included 78 patients from an area of high prevalence (>15%) of clarithromycin and levofloxacin resistant helicobacter pylori strains [1, 12, 14, 30, 31]. Both concomitant and bismuth-containing quadruple therapies are recommended as alternative first-line treatment according to the previously mentioned guidelines, particularly in regions with a high prevalence of clarithromycin resistance [13, 15].

The eradication rate for levofloxacin concomitant protocol was 82.05% according to ITT analysis, while doxycycline-based therapy had a PPA eradication rate of 78.94%, and 76.92% according to ITT analysis. The overall eradication rate of levofloxacin concomitant was about 3% higher than doxycycline-based therapy but the difference didn’t reach statistical significance. The result of levofloxacin containing therapy can be regarded as Grade D standard, while the result of bismuth-containing therapy can be regarded as Grade F as proposed recently [32]. The former results are better than our preceding study outcomes regarding the treatment of *H. pylori* [14]. Our results are agreeing with a meta-analysis by Essa et al. and similar research by Federico et al. Both studies showed that concomitant therapy is effective in the eradication of H.Pylori [26, 33]. Similarly, meta-analysis by Niv et al. found that the Doxycycline-based quadruple regimen had good efficacy in the treatment of Helicobacter Pylori infection [16] .Even though this research treated patients for a longer period [34], our results didn’t show the effectiveness mentioned in previous studies, this may be due to the existence of Helicobacter Pylori resistant strains [35]. Antibiotics are overused,especially macrolides and fluoroquinolones, for treating respiratory, urinary and gynaecological infections [36–38]. Chokshi et al. reported that the main contributors of emerging antibiotic resistance in developing countries are clinical mismanagement, antibiotics availability, poor quality of available antibiotics, and insufficient surveillance of resistance development [39]. We checked compliance by counting remaining pills which raised suspicions about outpatient incompliance to antibiotic regimens [40–42].

Our study has a few limitations (1) The only method to investigate medication history was to interrogate patients. Electronic medical were lacking, The patient was considered “naïve” if he/she wasn’t previously treated for *H. pylori* infection ;(2) We depended on patients to evaluate the compliance ;and (3) We didn’t perform a susceptibility test of *H. pylori* to antibiotics, because it’s unavailable in Syria.

This study showed that both regimens had an acceptable rate of eradication, and the difference didn’t reach statistical significance. These results were highly promising in treating *H. pylori* infection in Syria.

## Conclusion

The success rate of the standard triple therapy with clarithromycin or levofloxacin has declined substantially due to increasing antimicrobial resistance. (ACG) Clinical guideline and Maastricht Consensus recommended alternative regimens, including bismuth-containing quadruple therapy or non-bismuth concomitant therapy, as first-line therapies, particularly in areas with a high prevalence of clarithromycin resistance like Syria. Levofloxacin concomitant therapy wasn’t superior to doxycycline based quadruple therapy, we didn’t record any serious adverse event in both regimens. We encourage further researches to determine the optimal first-line empirical therapy for Helicobacter-Pylori Infection in the Syrian population.

## Data Availability

The dataset supporting the conclusions of this article is available for free in the [data.mendeley.com] repository, [https://data.mendeley.com/drafts/fzv77yzshx], and it will be available after 15 September 2021 to 15 September 2023.

## Abbreviations

PPA: Per-protocol analysis
ITT: Intention to treat analysis
q.i.d.: Four times a day
b.i.d.: Twice a day
q.d: Once a day
ACG: American College of Gastroenterology
*H. pylori*: Helicobacter Pylori
PPI: Proton pump inhibitors
